# From Fish Physiology to Human Disease: The Discovery of the NCC, NKCC2, and the Cation-Coupled Chloride Cotransporters

**DOI:** 10.34067/KID.0000000000000307

**Published:** 2023-11-16

**Authors:** Gerardo Gamba

**Affiliations:** Molecular Physiology Unit, Instituto de Investigaciones Biomédicas, Universidad Nacional Autónoma de México and Instituto Nacional de Ciencias Médicas y Nutrición Salvador Zubirán, Tlalpan, Mexico City, Mexico

**Keywords:** diuretics, thiazide, salt transport, hypertension, aldosterone, Bartter syndrome, BP, distal tubule, epithelial sodium transport, Gitelman syndrome, ion transport, kidney tubule, renal tubular epithelial cells

## Abstract

The renal Na-K-2Cl and Na-Cl cotransporters are the major salt reabsorption pathways in the thick ascending limb of Henle loop and the distal convoluted tubule, respectively. These transporters are the target of the loop and thiazide type diuretics extensively used in the world for the treatment of edematous states and arterial hypertension. The diuretics appeared in the market many years before the salt transport systems were discovered. The evolving of the knowledge and the cloning of the genes encoding the Na-K-2Cl and Na-Cl cotransporters were possible thanks to the study of marine species. This work presents the history of how we came to know the mechanisms for the loop and thiazide type diuretics actions, the use of marine species in the cloning process of these cotransporters and therefore in the whole solute carrier cotransproters 12 (SLC12) family of electroneutral cation chloride cotransporters, and the disease associated with each member of the family.

But without an internal environment of relatively constant composition in which complicated nerves, muscles, and glands could attain a high degree of elaboration and function in security, it is unlikely that the fresh-water fishes, with their elaborate sensory and motor equipment, would ever have been evolved. It was the evolution of the kidney that supplied the vertebrates with this stable body fluid. The most important constituents in the body fluid of all vertebrates are water and sodium chloride.

—Homer Smith, *From Fish to Philosopher. The Story of Our Internal Environment*

The Na-K-2Cl (NKCC2) and the Na-Cl (NCC) cotransporters are the major reabsorption pathways in the thick ascending limb of Henle loop (TAL) and the distal convoluted tubule (DCT), respectively.^1^ The genes encoding these transporters belong to the same superfamily of solute carriers, the SLC12, and exhibit a 50% identity at the amino acid level. NKCC2 and NCC share many physiological characteristics: Both are electroneutral cation-coupled to chloride transporters. They are regulated by intracellular chloride concentration and cell volume changes. They become active by phosphorylation of conserved threonine residues in the amino terminal domain, by a complex kinase system involving with no lysine kinases and the STE20-related/SPS1-related proline-rich/alanine-rich kinase and oxiative stress response kinase. These cotransporters are the receptors for the loop diuretics (NKKC2) and thiazide type diuretics (NCC) that are extensively used for the treatment of edematous states and arterial hypertension.

The diuretics inhibiting these cotransporters were invented long time before the mechanism of action was elucidated. The first real diuretic to be developed was the thiazide type diuretic, chlorthalidone. This drug was developed in 1957 in the Merck, Sharp, and Dome laboratories by a group of scientists that included James Sprague, Frederick Novello, and John Baer who synthetized chlorthalidone and conducted the animal tests that demonstrated its diuretic effect.^2^ Thiazides were the first useful pharmacological agent for the treatment of hypertension. Few years later, Karl Sturm in Germany introduced the furosemide, as a more potent diuretic than thiazides, and this was followed by bumetanide and ethacrynic acid. As mentioned, many years elapsed before the precise mechanism of action for the diuretics was discovered.

The mechanism of action of furosemide was first proposed to be due to the inhibition of active chloride reabsorption in the TAL by Malnic *et al*.^3^ Many years later, the work from Greger and Schlatter,^4,5^ simultaneous to that of Hebert and Andreoli,^6^ demonstrated that the mechanism for salt reabsorption in the TAL is due to the presence of a Na-K-2Cl cotransporter (NKCC2) coupled with potassium recirculation through an apical membrane potassium channel and that furosemide specifically inhibited this cotransporter (Figure 1). A similar cotransporter mechanism had been previously reported by Geck *et al.* in Ehrlich ascites cell that now we know is due to NKCC1,^7^ another Na-K-2Cl cotransporter, member of the SLC12 family, with 60% degree of identity with NKCC2.^1^

**Figure 1:**
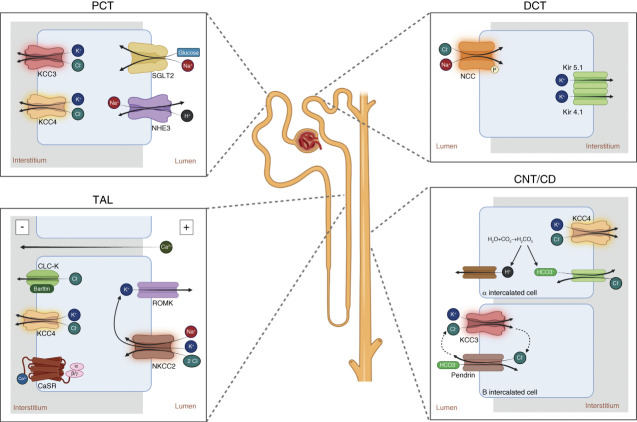
**The expression and role of cotransporters of the SLC12 family in the kidney.** KCC3 and KCC4 are expressed in the basolateral membrane. NKCC2 is present in the apical membrane driving the salt reabsorption and KCC4 in the basolateral membrane and participate in the KCl efflux. NCC is the salt reabsorption pathway. In the basolateral membrane heterodimers of Kir4.1 and Kir5.1 form the K^+^ efflux pathway. In the *α* intercalated cells KCC4 provides an efflux pathway for Cl^−^ allowing the continuous function of the Cl-HCO3 antiporter for the efflux of HCO3. In the B and non-A, non-B cells, KCC3 is present in the apical membrane functionally coupled with pendrin. In all cases the driven force for lumen to interstitum transport is due to the Na-K-ATPase located at the basolateral membrane, but it was not depicted to show mainly the role of the SLC12 transporters. CaSR, calcium sensing receptor; CNT/CD, connecting tubule and collecting duct. DCT, distal convoluted tubule; NCC, Na-Cl; NKCC2, Na-K-2Cl; PCT, proximal convoluted tubule; ROMK, renal outer medulla potassium channel; TAL, thick ascending limb.

The discovery of the mechanism by which thiazides produce their diuretic effect came from studies in fish. Early work by Diamond in the gallbladder of the fresh water roach (*Rutilus rutilus*)^8^ proposed for the first time the existence of an electroneutral Na-coupled to Cl mechanism for salt transport. Later, Renfro and colleagues analyzed the transport system in the urinary bladder of the sea water winter flounder (*Pseudopleuronectes americanus*) (Figure 2A).^9–11^ They used this model for two reasons. One is that in seawater fish, the renal system and urinary system must reduce the urine production at maximum to avoid dehydration, so salt reabsorption pathways should be expressed. The second reason is that in teleost, the urinary bladder is not homologous to that in amphibia or mammalians. The urinary bladder is a pouch that is contractile, but from mesonephric origin, it is not ectodermal. Thus, in teleost, the urinary bladder is anatomically and functionally like part of the mesonephric kidney.

**Figure 2:**
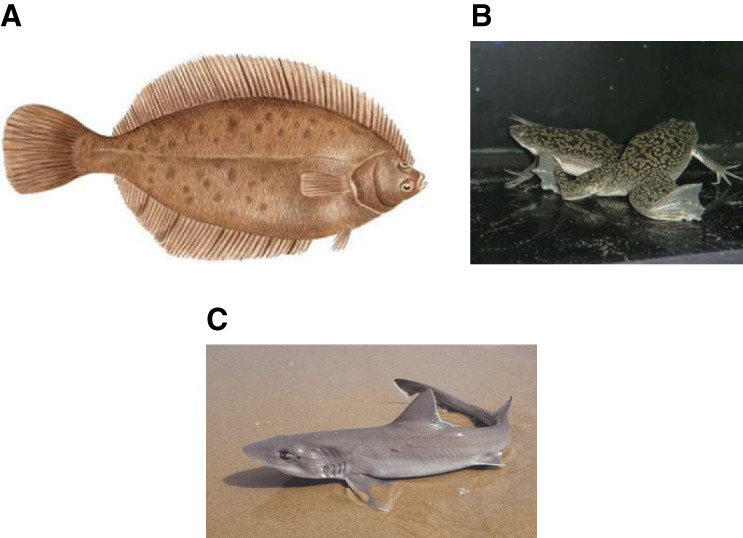
**Water living species that were key to identify the NCC and NKCC2 encoding genes.** (A) Winter flounder (Psuedopleuronectes americanus). (B) African frog (*Xenopus laevis*). (C)+ Dogfish shark (*Squalus acanthias*).

Renfro and colleagues observed that the flounder's urinary bladder possesses an apical Na-Cl transport system and its function is drive by a basolateral Na-K-ATPase.^9–11^ They also observed a Na^+^ uptake mechanisms that was independent of Cl^−^ but inhibited by amiloride, strongly suggesting that it is due to the epithelial sodium channel. Thus, the urinary bladder in the flounder seems to be like a DCT2 that is located outside the kidney. Years later, Stokes *et al.* for the first time explored the effect of hydrochlorothiazide on the NaCl transport system in the flounder urinary bladder.^12^ They observed that the NaCl transporter was potassium independent and that hydrochlorothiazide, but not furosemide, completely inhibited the NaCl absorption in a dose-dependent manner. This was the first study that unequivocally demonstrated the transport system that is inhibited by thiazides.

Some early studies suggested that thiazides site of action was the DCT,^13,14^ but it was until the work of Ellison *et al.* that clear evidence was presented, supporting the presence of a NaCl cotransport system in DCT that was specifically inhibited by thiazides^15^ (Figure 1). The mechanism by which this group of drugs induces natriuresis became clear almost 30 years after they were introduced in the market.

## The Molecular Identification of NCC

In 1990, I joined the Laboratory or Molecular Physiology and Biophysics at the Renal Division, Brigham, and Women's Hospital, Harvard Medical School, as a Fellow in Medicine, under the mentorship of Steven C. Hebert. The project I was assigned to work with was toward the cloning of the Na-K-2Cl cotransporter from the rat outer medulla, following a functional cloning strategy, using the African frog *Xenopus laevis* oocytes (Figure 2B). The cloning strategy using frog eggs^16^ had been perfectionated few years before by Hediger and Wright when they cloned the Na–glucose cotransporter, sodium glucose cotransporter 1.^17^

We started the project by injecting oocytes with mRNA extracted from the rat renal outer medulla because the Na-K-2Cl cotransporter is present in the medullary TAL. Initial results were encouraging, and we presented a free communication about it at the American Society of Nephrology Meeting in Washington, DC 1990, when this meeting was still held in a hotel. However, few weeks later, experiments started to fail and the signal we thought it was the renal Na-K-2Cl decreased and turned poorly reproducible. It was clear that renal outer medulla was not a good source of mRNA for functional expression of the renal Na-K-2Cl cotransporter, and because this cotransporter is no expressed elsewhere, as to search for another source of mRNA, our expectations of cloning the renal Na-K-2Cl cotransporter with the proposed methodology were seriously reduced.

**Figure 3:**
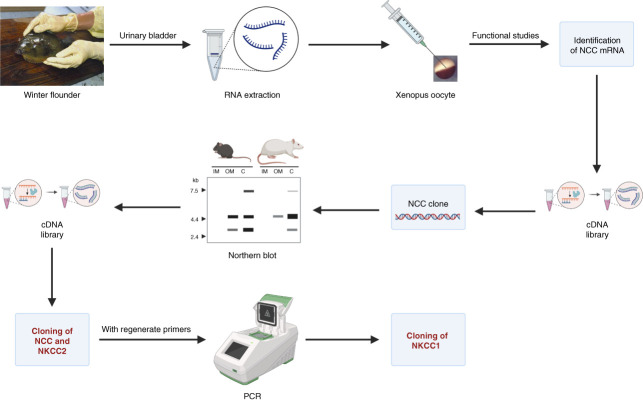
**The Hebert's laboratory approach for cloning the Na**^**+**^**-coupled chloride cotransporters.** mRNA was extracted from Winter flounder's urinary bladder was used for functional expression cloning of NCC in the heterologous expression system of *Xenopus laevis* oocytes.^18^ Then, flounder's NCC probe was used to clone both, NCC and NKCC2 from rat and mouse cDNA libraries.^19^ Finally, taking advantage that transmembrane 1 and ten segments of NCC and NKCC2 exhibited almost 100% identity, degenerate primers from these sequences were used for PCR to clone NKCC1 from an inner medullary collecting duct cell line.^20^

One morning at the beginning of 1991, while we were reviewing all the data we had, an idea that turned out to be brilliant came out. We thought that because the Na-K-2Cl and NaCl cotransporters had several functional properties in common, it was possible that they belonged to the same family, and if we cloned the NaCl cotransporter, we probably will be able to use that probe to pull out the Na-K-2Cl cotransporter cDNA form a rat or mouse outer medulla cDNA library (Figure 3). The proposal was that the winter founder's urinary bladder, having a similar transport system than the DCT, but being outside the kidney, was probably a good source of mRNA for the functional expression of the NaCl cotransporter because it was probably the only transporter in the apical membrane, while in the rat kidney, the DCT represents only a small fraction because glomerulus, proximal tubule, and blood vessels are much more prominent, and thus, the mRNA encoding the NaCl cotransporter would be diluted. Thus, to find the flounders, Steve and I went into 5 hours driving to the Mount Desert Island Biological Laboratory in Salisbury, Maine, in which Renfro and Stokes did their studies in the flounder and where the great Homer Smith used to go often to do experiments in marine species.

Back in Boston, as soon as I injected the mRNA extracted from the flounder's urinary bladder in the Xenopus oocytes, a robust expression of a Cl^−^-dependent Na^+^ uptake pathway that was inhibitable by thiazides, but not by furosemide, amiloride, or acetazolamide, was expressed. From there, it was a matter of time and hard work to identify a single 3.7 kb cDNA from a urinary bladder cDNA library encoding the NaCl cotransporter^18^ (Figure 3). This was the first member of the SLC12 family of electroneutral cotransporters to be identified at the molecular level.

## The Molecular Identification of Seven Members of the SLC12 Family of Electroneutral Cation-Coupled Cotransporters

Few weeks after cloning the NCC cDNA from flounder, a lot of excitement was produced when the Northern blot analysis of rat and mouse renal cortex and outer medulla at low stringency, using the flounder's cDNA probe, revealed a 4.5 kb bands from both, the cortex and medulla RNA (Figure 3). Knowing that the NaCl transporter was present only in DCT, that is, in the cortex, that observation indicated that the band in the cortex was certainly the mammalian NCC and the band in medullary RNA was most likely the transcript for the Na-K-2Cl cotransporter and that we will be able to identify the cDNA using the flounder's probe. And so it was. We were able to clone the mammalian cDNA encoding NCC and NKCC2 from both, rat and mouse kidneys^19^ (Figure 3), and later on, using degenerate primers constructed form NCC and NKCC2 identical sequences of transmembrane segments 1 and 10, we identified the cDNA encoding for the Na-K-2Cl cotransporter, NKCC1^20^ (Figure 3).

**Figure 4:**
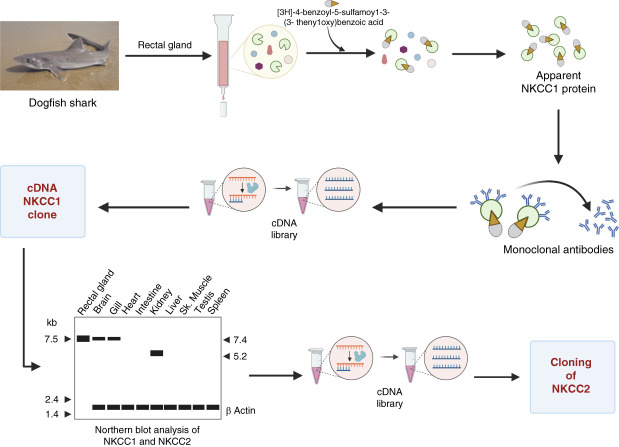
**The Forbush's laboratory approach for cloning NKCC1 and NKCC2.** Shark rectal gland proteins were subjected to separation of putative Na-K-2Cl cotransporter by [3H]-4-benzoyl-5-sulfamoy1-3-(3- theny1oxy)benzoic acid binding (a bumetanide-like photolabeling molecule). The isolated protein was then used to construct monoclonal antibodies that were used to screen a shark rectal gland cDNA library, to identify the NKCC1 cDNA.^21^ The, NKCC1 probes were used to clone NKCC2 from a rabbit renal cDNA library.^22^

The NKCC1 and NKCC2 cDNAs were identified at the molecular level simultaneously by the Hebert group at Harvard^19,20^ and the Forbush group at Yale.^21,22^ Forbush's group also started their projects by using a seawater species. In this case, they used the dogfish shark (Figure 2C) rectal gland in which they knew that a Na-K-2Cl cotransporter was prominent. Their strategy was to pull out proteins form the rectal gland that strongly bind to [^3^H]-4-benzoyl-5-sulfamoy1-3-(3- theny1oxy)benzoic acid, a photoreactive analog of bumetanide, and developed a monoclonal antibody against that protein that presumably was the cotransporter (Figure 4). This antibody was used to screen a shark rectal gland cDNA library from which a cDNA encoding NKCC1 was isolated.^21^ Using this probe, they identified both the NKCC1 and NKCC2 from mammalian sources^21,22^ (Figure 4).

The sequence of these transporters and the growing of the human genome project during the 90s allowed the *in silico* identifications of homologous mRNAs from the expressed sequence tags database that lead to the cloning of four genes encoding the K-Cl cotransporters, KCC1 to KCC4 that exhibit a 25% degree of identity with the Na-coupled transporters.^23–25^ Thus, the discovery of the SLC12 family genes was possible thanks to the initial cloning of NCC and NKCC1 from fish sources.

## Disease Associated with the SLC12 Family of Electroneutral Cotransporters

The identification of the cDNA of the SLC12 family members from fish and rodents opened the possibility to clone the cDNA from human sources and to search for the expected diseases to be caused by mutations in these genes and to construct knockout mice to explore the mechanism of disease (Figure 4).

### SLC12A1

**Figure 5:**
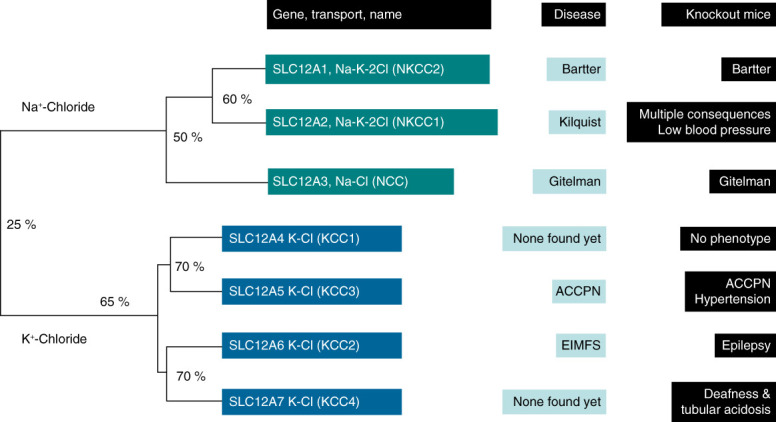
**Phylogenetic tree of the SLC12A family of electroneutral cation-coupled chloride cotransporters.** The name of the genes and transporters, the associated disease and the phenotype of knockout mice are depicted. Gamba G. *Membranes.* 2022;12:911.

Inactivating mutations in this gene encoding NKCC2 are the cause of the Barrter's syndrome type I, a hypokalemic metabolic alkalosis with low blood pressure (OMIM 601678)^26^ (Figure 5). When TAL or DCT activity is reduced, as in Bartter's or Gitleman's disease, respectively, the increased delivery of Na^+^ to the collecting duct increases the Na^+^ reabsorption by the epithelial sodium channel. This generates a negative voltage in the lumen that promotes the secretion of K^+^ by the apical potassium channel renal outer medulla potassium channel (ROMK). Thus, hypokalemia is produced. In addition, the increased amount of K^+^ in the collecting duct fluid, increase the activity of the K^+^/H^+^ pump that secretes H^+^ and thus, the loss of H^+^ in urine results in metabolic alkalosis.

Bartter's syndrome is genetically heterogenous.^27^ It can also be due to mutations in other ion transport pathways in the TAL whose inactivity also results in TAL failure. Type II, III and IV Bartter are due to inactivating mutations in the genes encoding the ROMK,^28^ the basolateral Cl^−^ channel chloride channel kidney B^29^ or the chaperon intracellular protein barttin,^30^ respectively (Figure 1). Because barttin is also a chaperone of the chloride channel kidney A channel, in addition to electrolyte renal wasting, these patients develop sensorineural deafness, because the impossibility to decrease intracellular chloride concentration in the stria vascularis cells, due to chloride channel kidney A and chloride channel kidney B inactivity. This results in reduction of the basolateral NKCC1 activity and thus, no potassium is available for secretion into the endolymph.^31,32^

Type V Bartter is a transient form of antenatal Bartter-like syndrome presented clinically as polyhydramnios. This disease has been linked with mutations in the gene encoding the melanoma associated antigen D2 that is mapped to chromosome X.^33^ This melanoma associated antigen D2 protein affects the expression and function of NKCC2, possibly through a cAMP related mechanism.

Another Bartter-like syndrome is produced by activating mutations of the calcium sensing receptor (CaSR).^34,35^ When extracellular calcium raises, the activation of the G*α*_q_ CaSR in the basolateral membrane of the TAL (Figure 1), reduces the activity of the Na-K-2Cl, NKCC2 and the apical K^+^ channel, ROMK, to reduce the intraluminal positive voltage that drives the Ca^2+^ reabsorption trough the paracellular pathway (Figure 1), thus inducing hypercalciuria. Activating mutations in the gene encoding the CaSR results in a receptor that is active under normal or even low extracellular Ca^2+^ concentration that keeps the TAL activity reduced, producing a Bartter-like disease.

### SLC12A2

Inactivating mutations of this gene encoding NKCC1 brings multiple consequences. NKCC1 is expressed in the basolateral membrane of all secretory epithelia, in all nonepithelial cells in which its function is critical for cell volume regulation and in neurons in which its activity modulates the intracellular chloride concentration and thus, the effect of neurotransmitters acting in Cl^−^ channels in the post synaptic membranes. Loss of function mutations of NKCC1 have been associated with an extremely rare autosomal recessive disease known as Kilquist syndrome (OMIM 619080)^36^ (Figure 5) exhibiting sensorineural deafness, because the absence of NKCC1 in the basolateral membrane of the stria vascularis cells in the inner ear alter the quality of the endolymph, intestinal and respiratory disfunction, since the absence of NKCC1 in basolateral membrane of secretory epithelia reduce the chloride to be secreted by the cystic fibrosis transmembrane regulator channel, neuropsychological delay, probably due to the change of the gama aminobutric acid (GABA) effect in some neuronal circuits, and severe xerostomia, because of the absence of saliva secretion. A 13 year old girl with multisystemic disfunction characterized by orthostatic intolerance, respiratory weakness, multiple endocrine abnormalities, pancreatic insufficiency, and multiorgan failure involving the gut and bladder was found to have a deletion of 11 base pairs in one allele of NKCC1 that precluded its function.^37^ One report associates an autosomal dominant gain of function mutation of NKCC1 in a patient with schizophrenia.^38^

### SLC12A3

Several loss of function mutations in the gene encoding NCC have been associated with the development of Gitelman's syndrome, also characterized by hypokalemic metabolic alkalosis with low blood pressure (OMIM 263800)^39,40^ (Figure 5). It is usually less severe than the Barrter's syndrome and frequently diagnosed until the adolescence. One distinct feature between Bartter and Gitelman is that the first exhibits hypercalciuria, since in the TAL the salt and calcium reabsorption are associated, while the second exhibits hypocalciuria since the inactivation of the DCT is associated with increased calcium reabsorption.

A Gitleman-like disease is also present in mutations of mitochondrial DNA^39^ and in a rare and complex neurological disease known as SeSAME (OMIM 612780) exhibiting sensorineural deafness, seizures, ataxia, mental retardation, and electrolyte disturbances.^41,42^ This disease is due to mutations in the gene encoding the potassium channel Kir4.1 that is present in the DCT basolateral membrane (Figure 1). Inactivation of this channel results in depolarization of the DCT cell that is compensated by increasing the intracellular chloride concentration, which in turn, reduces the activity of NCC, resulting in a Gitelman-like syndrome.^43^

### SLC12A4

This gene encodes for the K-Cl cotransporter, KCC1. This member of the family is expressed in many cells in several tissues and like the other KCCs its function is to provide a pathway for K-Cl efflux of the cell. The major role of KCC1 is the regulation of cell volume. In isotonic conditions is inhibited and is activated during cell swelling to reduce the intracellular ion concentration, as part of the regulatory volume decrease mechanism. No disease has been associated with KCC1 mutations and the knocking out of this cotransporter in mouse results in no phenotype (Figure 5). It is possible that the absence of KCC1 is compensated by the other KCCs cotransporters.

### SLC12A5

KCC2 is a central nervous system specific gene. It is only expressed in neurons. KCC2 is a unique K-Cl cotransporter that is active in isotonic conditions, although like the other KCCs, it can be further activated by hypotonicity.^44^ Its presence and activity in neurons are fundamental to provide a pathway for Cl^−^ efflux and thus to promote a low intracellular chloride concentration.^45^ This condition is necessary for neurotransmitters like the GABA to perform as inhibitory neurotransmitter. The interaction of GABA with its receptor opens a Cl^−^ channel. If the potential equilibrium for Cl^−^ in the synaptic neuron is negative, then the opening of Cl^−^ channels results in hyperpolarization of the neurons due to the entrance of Cl^−^, following its gradient. Thus, a neurotransmitter like GABA can be excitatory or inhibitory, depending on the potential equilibrium for Cl^−^.

Complete knockout of KCC2 in mice results in neonatal death due to inability to breath.^46^ No neurogenic stimuli are produced for ventilation. With 95% reduction of KCC2, the mice can breathe, but develops and almost continuous epilepsy and died in the first 17 days. In humans, inactivating mutations in KCC2 have been associated with autosomal dominant idiopathic epilepsy (OMIM 616685) and with autosomal recessive epilepsy of infancy with migrating local seizures (OMIM 616645)^47^ (Figure 5).

### SLC12A6

KCC3 is expressed in many tissues, including the central nervous system. In the kidney it is expressed in the basolateral membrane of the proximal tubule.^48^ Its role, has been proposed to be a pathway for K-Cl efflux during cell swelling due the intense activity of the Na–glucose (sodium glucose cotransporter 2) transporter. The extraction of K^+^ is required to keep the Na^+^-K^+^-ATPase of the basolateral membrane working at maximum. In this regard, the expression of KCC3 in proximal tubule is increased during hyperglycemia.^49^ KCC3 is also expressed in the apical membrane of the intercalated pendrin-expressing cells in which it's been proposed to be important for the response to metabolic alkalosis.^50,51^

Loss of function mutations in KCC3 results in a complex neurological hereditary disease with motor and sensory neuropathy, associated with agenesis of the corpus callosum, also referred as Anderman's disease (OMIM 218000)^52^ (Figure 5). The syndrome has its higher incidence in Quebec, Canada due to a founder effect back in 17th century. The knockout mice recapitulate the neurological disease and is also hypertensive. Although the mechanism for hypertension has not been clarified, it could be due to a vascular effect.

### SLC12A7

KCC4 is also expressed in many tissues. In the kidney it is present in the basolateral membrane of the proximal tubule, the TAL and the intercalated cells (Figure 1). No human disease has been associated with mutations in KCC4, but the knockout in mice results in sensorineural deafness and renal tubular acidosis^53^ (Figure 5).

## Concluding Remarks

The cloning of the genes encoding the whole SLC12A family of electroneutral cation chloride cotransporters was possibly thanks to physiological studies and cloning of the first members (NCC and NKCC1) from seawater species. Mutations in members of this family are the cause of at least six human inherited diseases of the cardiovascular, renal, and central nervous system and, most likely, are involved in the genetics of complex diseases such as arterial hypertension, epilepsy, and osteoporosis. As proposed by Homer Smith in his book form From Fish to Philosopher, the study of fish physiology has beneficiated the development of knowledge in human physiology and pathophysiology.
